# PeakCaller: an automated graphical interface for the quantification of intracellular calcium obtained by high-content screening

**DOI:** 10.1186/s12868-017-0391-y

**Published:** 2017-10-16

**Authors:** Elena Artimovich, Russell K. Jackson, Michaela B. C. Kilander, Yu-Chih Lin, Michael W. Nestor

**Affiliations:** 1 The Hussman Institute for Autism, Baltimore, MD USA; 2 Hussman Strategic Advisors, Ellicott City, MD USA

**Keywords:** Calcium imaging software, High-content screening, Human stem cells, Calcium imaging

## Abstract

**Background:**

Intracellular calcium is an important ion involved in the regulation and modulation of many neuronal functions. From regulating cell cycle and proliferation to initiating signaling cascades and regulating presynaptic neurotransmitter release, the concentration and timing of calcium activity governs the function and fate of neurons. Changes in calcium transients can be used in high-throughput screening applications as a basic measure of neuronal maturity, especially in developing or immature neuronal cultures derived from stem cells.

**Results:**

Using human induced pluripotent stem cell derived neurons and dissociated mouse cortical neurons combined with the calcium indicator Fluo-4, we demonstrate that PeakCaller reduces type I and type II error in automated peak calling when compared to the oft-used PeakFinder algorithm under both basal and pharmacologically induced conditions.

**Conclusion:**

Here we describe *PeakCaller*, a novel MATLAB script and graphical user interface for the quantification of intracellular calcium transients in neuronal cultures. *PeakCaller* allows the user to set peak parameters and smoothing algorithms to best fit their data set. This new analysis script will allow for automation of calcium measurements and is a powerful software tool for researchers interested in high-throughput measurements of intracellular calcium.

## Background

Intracellular calcium ([Ca^2+^]) regulates key neuronal cell cycle and signaling pathways, including development and proliferation, axon guidance, presynaptic neurotransmitter release, and postsynaptic activity-dependent synaptic plasticity [1–4]. Dysregulation of [Ca^2+^] concentration has been associated with multiple neurodevelopmental, neuropsychiatric, and neurodegenerative diseases, including Alzheimer disease, autism spectrum disorder, and schizophrenia [5–9]. For extensive reviews of the function of Ca^2+^ in neuronal cells please see [10, 11]. The combination of Ca^2+^ imaging with the use of human induced pluripotent stem cells (hiPSCs) provides a very powerful toolset for interrogating the role of subsets of neurons and neural circuits in patient-specific disease models. Besides describing the basal function of hiPSC-derived neurons, Ca^2+^-based imaging can test the hypothesis that hiPSC-based models accurately recapitulate the genetic background and early developmental physiology of the individuals from whom they are harvested [8, 12–14].

The advent of high-throughput imaging technology, such as ThermoFisher’s Array Scan platform (ThermoFisher Scientific Waltham, MA, USA), combined with the use of hiPSC-based methods can generate large amounts of raw Ca^2+^ imaging data. Critical to accurate measurements of cellular Ca^2+^ is the analytics platform through which the raw data is processed. There are currently a number of high-powered Ca^2+^ analysis programs that have a wide breadth of functionality addressing this problem (Table 1). For example, FluroSNNAP is a program that allows for semi-automated analysis of Ca^2+^ transient event detection [15]. FluroSNNAP also has the ability to use local and global synchronization analysis to identify related neurons in a network and graph functional connectivity. In order to assure unbiased analysis of cellular Ca^2+^ spikes, [16] developed a MATLAB based program that focuses on single and multiple transient responses by giving quantifiable parameters for time of onset after stimulus application, amplitude of response, response duration, area under the curve, and decay time constant among other measurements. For looking at large number of cells, NeuroCa is able to process ~ 1000 Ca^2+^ spikes from a given recording field. This software can allow for the mapping of functional connectivity of large networks of cells by mapping the spatiotemporal Ca^2+^ spike patterns amongst the network [17]. Another MATLAB-based program developed by [18] is designed to distinguish individual cells in densely populated cultures and measure their Ca^2+^ activity. The program does this using a segmentation algorithm combined with time-series analysis to measure Ca^2+^ transients. Finally, SIMA is a Python-based open source software package designed for 2-photon Ca^2+^ imaging experiments. SIMA is able to efficiently correct for motion artifacts as well as background noise while applying segmentation and extraction of Ca^2+^ transients from in vitro and in vivo tissue [19, 20].

**Table 1:** Comparison of software packages that measure intracellular calcium activity

Software package	Major features of calcium dynamics	References
SIMA	Amplitude Time of onset Area under the curve Frequency Activation and decay time	[19, 20]
FluoroSNNAP	Amplitude Functional connectivity Synchronization analysis Correlation coefficient Frequency Activation and decay time Wavelet-based detection	[15]
NeuroCa	Amplitude Frequency Background correction Analysis of ~ 1000 cells Network dynamics	[17]
PeakCaller	Frequency Synchronization index Correlation coefficient Event rise and fall time	
MATLAB dynamics	Amplitude Frequency Time of onset after stimulus Area under the curve Response duration Full width half max Decay time constant	[16]
MATLAB script	Amplitude Frequency Automated detection of single cells in dense cultures Background correction	[18]

There are a number of software packages designed to measure Ca^2+^ transients in cells, and each program has strengths and weaknesses. Nonetheless, many research labs still use the PeakFinder MATLAB script to do simple peak detection of fluorescent signals from cell populations. In light of having many options for the researcher to choose from when analyzing Ca^2+^ activity in neurons, we have developed *PeakCaller*-an enhanced version of *PeakFinder*.

*PeakFinder* is a MATLAB script which automates the identification of “peaks” of independent variables over time, and is commonly used in the quantification of Ca^2+^ signaling. However, *PeakFinder* has several limitations that can result in an increase in type I and type II errors. To address these limitations, our group has developed *PeakCaller*, a MATLAB GUI interface and algorithm. In the following manuscript, we present empirical evidence of the efficiency of *PeakCaller* by demonstrating how the use of *PeakCaller* reduces the occurrence of type I and type II errors. We test this hypothesis across multiple Ca^2+^ indicators and analysis platforms. Finally, we provide a step-by-step guide to using *PeakCaller*, and a detailed justification of the algorithms used in *PeakCaller*.

## Implementation

### hiPSC-derived cortical neuron cultures

HIP™ Neural Stem Cells (MTI-GlobalStem, Gaithersburg, MD) were cultured according to the manufacturer’s specification. Cells were cultured in GlobalStem Expansion Media (Neurobasal medium)(Life Technologies) supplemented with 2 mM Glutamax (Gibco), 2% B-27 supplement (Gibco), 1X non-essential amino acids (Gibco), and 20 ng/mL FGF-2 on 1:200 Geltrex-coated 6 well culture dishes and incubated at 37 °C/5% CO_2_. After a 7–10 day recovery period, HIP™ Neural Stem Cells were disassociated from the culture dishes using pre-warmed Accutase (Gibco) and plated at 120,000 cell/well onto Perkin-Elmer 24 well glass-bottom culture plates. Plates were coated with 1X polyornithine (Gibco) for 1 h at 37 °C/5% CO_2_ and 1:333 Laminin (Life Technologies) for 24 h at 37 °C/5% CO_2_. The cells were directed toward a neuronal fate using Global Stem maintenance media (NeuralQ Basal Medium, GlobalStem), 2% GS21 Supplement (GlobalStem), and 0.5 mM l-Alanine/l-Glutamine. Three-quarters media change was performed every other day. Cells were kept in these culture conditions until day 30 post differentiation.

### Dissociated mouse cortical neuron culture

Cortices from postnatal day 0–0.5 (P0-0.5) mice (C57BL/6J) were dissected out according to procedure described in [21]. Tissue was dissociated for 70–90 min at 37 °C using 10 U/mL Papain (Worthington, Lakewood, NJ) in HBSS containing 0.2 mg/mL l-cysteine (Sigma), 1 mM Pyruvate, 10 mM HEPES, 100 U/mL Penicillin, 100 mg/mL Streptomycin. Thereafter, dissociated tissue was rinsed twice in HBSS solution, with an addition of 10 U/mL DNase (Sigma) in the first wash, and twice in NB-FBS (Neurobasal A medium supplemented with 2% B-27 supplement, 2 mM l-glutamine, 100 U/mL Penicillin, 100 mg/mL Streptomycin and 10% FBS) (Sigma). To obtain a single cell suspension, tissue was triturated in NB-FBS using a P1000 pipette. For Ca^2+^ imaging experiments, cells were plated onto glass bottom Fluorodishes (World Precision Instruments, Sarasota, FL) coated with 20 mg/mL poly-d-lysine in 20 mM HEPES solution, at a concentration of 1.5 × 10^6^ cells/mL in NB-FBS. Plating medium was exchanged to serum-free NB culture medium after 3–15 h. Cells were maintained in culture for 14 days in vitro (DIV) at 37 °C/5% CO_2_. All cell culture reagents were from Gibco/Invitrogen (ThermoFisher) unless otherwise stated.

### Fluo-4 Ca^2+^ Assay and pharmacological interrogation

Both hiPSC-derived cortical neurons and dissociated mouse cortical neurons were subjected to the Fluo-4 Direct Ca^2+^ Assay (ThermoFisher). Assay reagents were prepared according to manufacturer’s protocol and Fluo-4 was added 1:1 with cell culture media. Cells were incubated at 37 °C/5% CO_2_ for 30 min prior to imaging. Cells were imaged using the Zeiss Spinning disk confocal microscope. Cells were recorded for 10 min at 1 frame every 600 ms (1000 frames total) at 488 nm. Zen Blue software was used to manually designate ‘Regions of Interest’ (ROI) around cell bodies that showed active Ca^2+^ flux. Raw mean fluorescent data was exported via *.txt file for each ROI. To test how the *PeakCaller* script would perform under pharmacological conditions resulting in increased neuronal excitation as compared to *PeakFinder*, we exposed hiPSC-derived cortical neuronal cultures to Fluo-4 for 30 min then added 50 μM of the GABA_A_ channel inhibitor picrotoxin (PTX) (Tocris) to the culture media. Cells were imaged for 40 min (4 × 10 min recordings) and ROIs were selected as described above. The same ROIs (cell bodies) were traced for the duration of the assay. Where indicated, some human iPSC-derived neurons were incubated with Adeno-associated Virus containing vectors for the Ca^2+^ indicator GCamp6 (AAV-GCamp6, University of North Carolina Vector Core). After incubation, neurons were subjected to imaging using a Zeiss LSM Duo at a rate of 10 Hz (University Of Maryland, School Of Medicine Confocal Microscopy Core).

In order to determine whether the variable parameters and smoothing features of *PeakCaller* functioned as designed in reducing the incidence of type I and type II errors, we compared the rate of type I and type II errors in commercially available hiPSC-derived cortical neurons (d30 post differentiation) and dissociated mouse cortical neurons. In this instance, type I and type II errors were determined manually by the user. For interpretation of the results type I error is defined as the probability of rejecting the null hypotheses when the null hypotheses is statistically true (false positive). The type II error is incorrectly accepting the null hypotheses when the null hypothesis is statistically false (false negative) [22, 23]. Representative Ca^2+^ traces are presented to demonstrate what was called a type I and type II error with each experiment. Significance was determined by Chi square statistic, with an alpha of 0.05.

*PeakCaller* is a MATLAB-compatible script developed to allow users greater control over peak parameters and to apply data smoothing algorithms designed to reduce type I and type II errors in Ca^2+^ signaling studies in cultured neurons. This script and additional information can be found at www.hussmanautism.org/resources/software. While developed and applied to Ca^2+^ imaging in this study, *PeakCaller* can be used for any data set in which there is a need to detect the number and frequency of peak changes in the dependent variable over time. Other applications include financial data, solar emissions, and epidemiological modeling. In the instance of Ca^2+^ signaling, a “peak” represents the rapid increase of intracellular Ca^2+^ needed to trigger disparate cellular signaling cascades. At the cellular level, the raw data for *PeakCaller* can be collected in multiple ways (for a detailed review of Ca^2+^ signaling studies and Ca^2+^ indicators see: [24]). In most instances the raw data output from these various methods is similar: a time-dependent change in the intensity of the fluorescence signal within a defined ROI.

Whether performed on a traditional confocal microscope like the Zeiss Spinning Disk, or an automated high-throughput system like the ThermoFisher Array Scan, the user will specify a ROI within the recorded field (in the case of a neuron, typically a selected cell body or dendrite). The average fluorescence intensity of the pixels within the ROI will be recorded for each time point and can be exported according to the software used by the recording platform. *PeakCaller* is designed to automate the process of determining the number and frequency of Ca^2+^ signaling events (as estimated by temporal increases in the mean fluorescence per ROI) in an intuitive manner. In addition to analyzing Ca^2+^ signaling data, *PeakCaller* has an additional useful function particular to high-throughput systems like the Array Scan platform. *PeakCaller* will remove from the analysis incomplete data sets, which can arise when a cell body is dropped from the recording due to a significant decrease in the fluorescent signal measured during the automation process.

*PeakCaller* is easy to run and provides a simple way to visualize results, set appropriate analytics parameters, and record results in a concise and convenient format. When *PeakCaller* is started, only a single button labeled “Choose File” is active. Clicking this button opens a standard dialog box that prompts the user to pick a data file for analysis. For this manuscript, two imaging systems, the Zeiss Spinning Disk Confocal (Carl Zeiss AG, Oberkochen, Germany) and the ThermoFisher Array Scan (ThermoFisher Scientific Waltham, MA, USA) were used to generate the datasets used in the analysis; these datasets were saved as comma-delimited files (*.csv). In addition to being the two major platforms used for obtaining cell-based Ca^2+^ signaling, the Zeiss platform represents a low-to-medium throughput system, whereas the ThermoFisher system represents a high-throughput system. These two different systems produce data in two different formats. *PeakCaller* makes a quick check of the formatting to determine which type of data is present. (Examples of the different datasets are included on the website along with *PeakCaller*. Readers are encouraged to consult the Zeiss Zen Blue and Array Scan HCS Studio 2.0 software for detailed information of performing Ca^2+^ signaling experiments.)

Once a data set is successfully loaded, the software displays a button labeled “Find Peaks,” along with a panel that allows the user to fine-tune the qualities they require of peaks in their data. First, the user can choose to remove a background trend from the data, using the methods detailed in the section “*PeakCaller*–Smoothing Features.” Second, the user can define the characteristics that a peak should have: how far and how fast the data must rise and fall for a point to be characterized as a peak, as noted in the section “*PeakCaller*–Paramterizing a Peak.” These two key features are major improvements over the simpler *PeakFinder* MATLAB script, in that they allow the user more control over peak definition, and they adjust for global time-based changes in the intensity of the fluorescent signal that may occur during the course of an experiment, such as photobleaching.

Once the user has selected the parameters that best represent their data, they click the “Find Peaks” button and the results are displayed visually on two graphs–the upper graph shows the original data and the defined long-term trend, while the lower graph shows the de-trended data. Each has all identified peaks marked with small green circles. A scroll bar below the graphs allows the user to quickly see the results for all regions of interest included in the data file.

The peak data is also displayed in the MATLAB command window, and the user can choose to extract the lower graph (for easy manipulation and printing) or save the peak data to a file. For each region of interest, the software displays the number of events (peaks), the intervals between events, and the average frequency implied by those intervals. It also provides the average frequency of events across all ROIs, the mean number of events per ROI, and the standard deviation of the number of events per ROI.

### PeakCaller: smoothing features

*PeakCaller* offers several tunable options for muting the effects of an underlying trend in a time series. Minimizing the influence of such trends can be important when there are global changes in background fluorescence due to bleaching or changes in focus that cause the measured baseline fluorescence to become nonlinear. *PeakCaller* approximates the underlying trend by generating a smoothed version of the time series, which emphasizes the long-term movements in the time series and deemphasizes the quick peaks and background fluctuations. *PeakCaller* includes four options for generating this smoothed series: the first three choices might be considered more traditional convolution-based smoothers, while the fourth has a more topological flavor. In any case, the resulting smoothed version of the time series serves as a rough approximation of the underlying trend, and *PeakCaller* controls for this trend simply by dividing the original time series by the smoothed profile. The four options for generating this smoothed background trend line are described here.

#### Finite difference diffusion

**Fig. 1:**
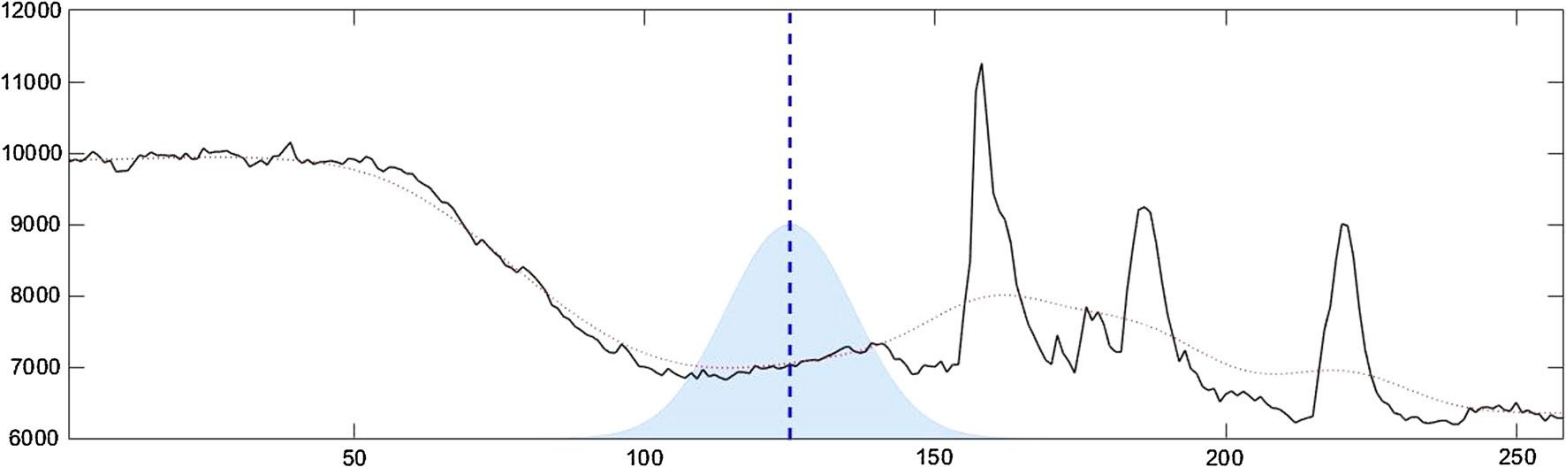
This figure illustrates the weight function and smoothing corresponding to finite difference diffusion, with the trend smoothness parameter set to 80. Time is on the horizontal axis (in seconds) and the fluorescence intensity of the Fluo-4 emission is on the vertical axis (arbitrary units). For the original Ca^2+^ recording (in black), the smoothing is given by the dotted red curve. To visualize the smoothing calculation, at the dashed blue line, the smoothed value is computed by weighting the values of the original profile proportionally with the weight function shaded in light blue. Notice that this weight function is approximately Gaussian

In physical systems, the process of diffusion describes the collective motion of particles. Real-world examples of diffusion include things like the movement of milk that has been poured into a cup of coffee or the spread of engine exhaust from the tailpipe of a car. Over time, particles move from areas of high-concentration to low, and everything becomes more and more uniform. In *PeakCaller*, finite difference diffusion numerically approximates this process—treating the original time series much like an initial measure of the spatial concentration of engine exhaust—and numerically approximating its diffusion and spread. Small values of the parameter trend smoothness correspond to short diffusion times (less smoothing), whereas larger values correspond to longer diffusion times (more smoothing). Figure 1 shows a representative recording from a hiPSC-derived neuron treated with Fluo-4. In this case, the smoothed profile shown was produced via finite difference diffusion. Additionally, the figure includes an overlay showing how the values of the original profile are weighted in order to generate the smoothed profile.

#### Exponential moving average (1-sided)

**Fig. 2:**
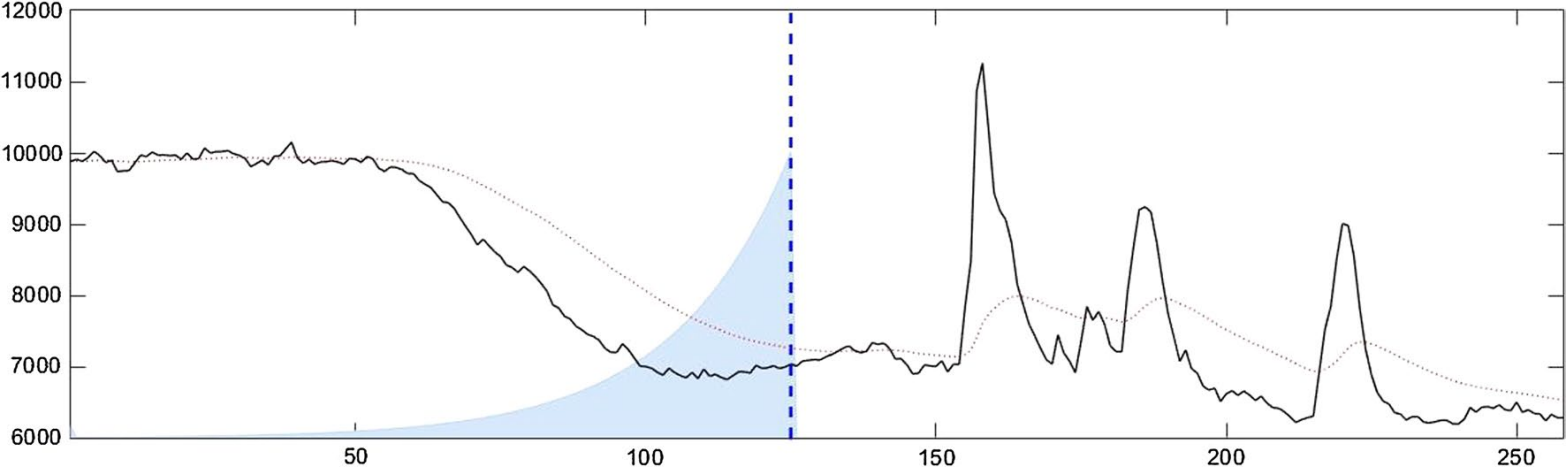
Example of  the weight function and smoothing corresponding to a 1-sided exponential moving average with the trend smoothness parameter set to 40. For the original Ca^2+^ recording (in black), the smoothing is given by the dotted red curve. To visualize the smoothing calculation, at the dashed blue line, the smoothed value is computed by weighting the values of the original profile proportionally with the weight function shaded in light blue. Note especially that the smoothed value at any point depends only on the portion of the original profile to the left of that point

Moving averages provide an elementary technique for smoothing time series, and are often used in finance to highlight long-term trends. For any point in the data set, the exponential moving average depends only upon the data up to that point, with the weighting of the older data falling off exponentially. In *PeakCaller*, small values of the parameter trend smoothness correspond to fast-decaying exponentials (less smoothing), whereas larger values correspond to longer decay times (more smoothing). Figure 2 shows a representative recording from a hiPSC-derived neuron treated with Fluo-4. The smoothed profile was produced using an exponential moving average. Additionally, the figure includes an overlay demonstrating how the values of the original profile are weighted in order to generate the smoothed profile.

In practice, this method is particularly useful for data sets with a higher degree of changes in background fluorescence, due to conditions such as overgrown (multi-layered) cell cultures, cultures with an excess degree of dead cells, or weak Ca^2+^ signals that may be hard to distinguish from background. This method is recommended when changes in culture conditions over time are expected due to pharmacological intervention, which could affect the characteristics of future peaks.

#### Exponential moving average (2-sided)

Exponential moving averages are commonly used with live data, where only the past history is known; an estimate of the current trend cannot make use of future data that is not yet in evidence. In the current context however, we already have the entire recording of Ca^2+^ transients from a given time window, and thus can approximate the trend at any point by using measurements taken both before and after that particular moment in time. One option is to simply average a backward-looking exponential moving average (looking back to the beginning of the recording) with a forward-looking exponential moving average (looking forward to the end of the recording). This method might be recommended over the classic exponential moving average (1-sided) when culture conditions remain static.

**Fig. 3:**
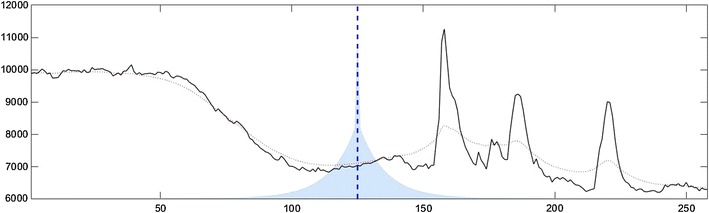
Graphical representation of  the weight function and smoothing corresponding to a 2-sided exponential moving average with the trend smoothness parameter set to 20. For the original Ca^2+^ profile (in black), the smoothing is given by the dotted red curve. To visualize the smoothing calculation, at the dashed blue line, the smoothed value is computed by weighting the values of the original profile proportionally with the weight function shaded in light blue. Notice especially the double-weight given to the value at the blue line, as it is the only point in common to both the forward- and backward-looking moving averages

Figure 3 shows a representative recording from a hiPSC-derived neuron treated with Fluo-4 and the smoothed profile produced using the 2-sided exponential moving average. Additionally, the figure includes an overlay showing how the values of the original profile are weighted in order to generate the smoothed profile. Note that these weights differ from the other two-sided weights given via finite-difference diffusion in Fig. 1.

#### Convex envelope

**Fig. 4:**
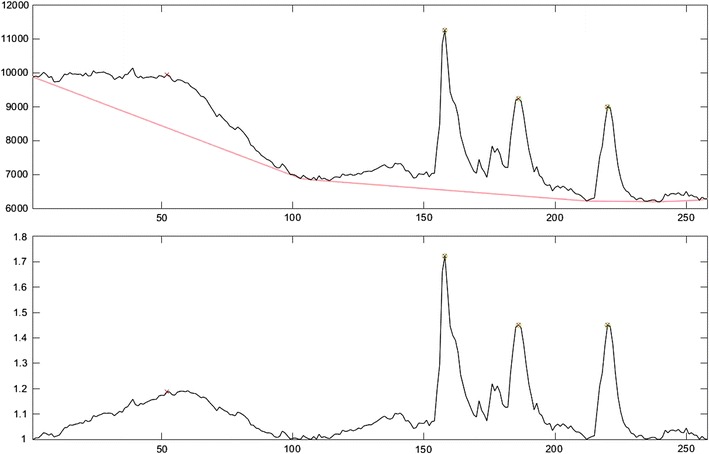
Illustration of smoothing using the convex envelope and shows the actual graphics provided by *PeakCaller*. In the upper graph, for the original Ca^2+^ recording (in black), the smoothing using a convex envelope is given by the dotted red curve. The lower graph shows the de-trended data series, which is simply the quotient of the original data divided by the smoothed data. Note in this case that the de-trended data “bounces off” of a minimum value of one exactly at the corners where the convex envelope touches the original profile. Peaks identified by *PeakCaller* are marked with a green circle, whereas peaks identified by *PeakFinder* are marked with a red ‘X’

This is a purely geometric measure that defines the trend as the portion of convex envelope underneath the graph of the time series. Intuitively, one can picture an elastic band running under the graph and tied to both end points. When that band is snapped tight along the bottom, the resulting convex envelope can be used as a measure of the trend. In *PeakCaller*, the parameter trend smoothness plays no role in defining the trend in this case, as the smoothing is defined in a parameter-free way by an advanced game of “connect the dots”. The smoothing and the resulting de-trended profile are both displayed in Fig. 4.

#### No trend

In instances where the original data is linear in nature and no change to culture conditions is suspected, there is an option to leave the raw data untransformed. When no trend is chosen, the mean of the original profile is used as a trivial smoothing. The de-trended profile displayed by *PeakCaller* will then have exactly the same shape as the original profile, but scaled so that the mean is 1.

### *PeakCaller*: parameterizing a peak

*PeakCaller* characterizes peaks using a clear and straightforward set of criteria. In order for a point to be called a peak, the de-trended data must rise a significant amount in a well-defined window just before the candidate point. The user can define the size of the rise that they deem significant (by setting required rise (%) in *PeakCaller*) as well as the maximum length of the window allowed for the rise to occur (max look-back (pts) in *PeakCaller*). The actual look-back window (the time interval in which the florescent must rise in order to be called a peak) will be shortened appropriately if either (a) the max look-back goes beyond a previously identified peak or (b) the max look-back goes beyond the beginning of the data.

In complementary fashion, for a point to be a peak, the de-trended data must fall a significant amount in a well-defined window following the candidate point. The user can define the size of the fall that they deem significant (by setting required fall (%) in *PeakCaller*) and the maximum length of the window allowed for the fall to occur (max look-ahead (pts) in *PeakCaller*). The actual look-ahead window will be shortened appropriately if either (a) the max look-ahead goes beyond the end of the data or (b) the max look-ahead includes points that are higher than the candidate.

**Fig. 5:**
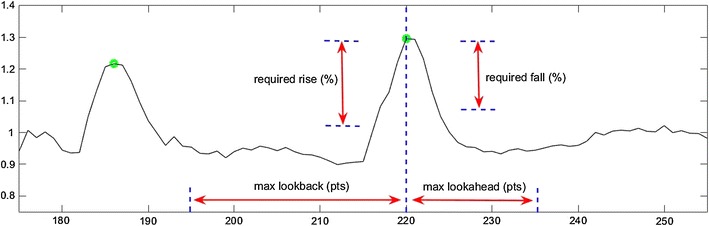
This figure details the parameters used by *PeakCaller* to characterize a peak. For a point to be classified as a peak there must be a significant rise over a designated interval before the point, and a significant fall within a designated interval after the point

A diagram showing the four quantities that a user may use to define a peak is given in Fig. 5. Although many users may choose matching values for rise and fall, as well as look-back and look-ahead, *PeakCaller* uncouples these values to allow the user some versatility. In many applications, signals are not perfectly symmetric–with depolarization and repolarization happening on different timescales–and the user now has the freedom to vary these parameters as they deem appropriate.

*PeakCaller*’s GUI allows users without MATLAB coding experience to modify peak parameters to best interpret their data. The features described above coupled with the GUI are intended to be an improvement over the original *PeakFinder* algorithm. *PeakFinder* classifies peaks according to an approximation for the area of candidate peak formations. This scoring for peaks rewards the wide candidate peak formations in a way that is not always appropriate in the search for quick localized spikes. Also, in developing cortical neuron cultures, Ca^2+^ peaks may not have a time scale as well developed as that in dissociated mouse cortical neurons. The ability to set culture-specific look-back windows is intended to allow the user to modify the peak calling script to suit the developmental state of their cultures. The *PeakFinder* script was not originally designed to process Ca^2+^ signaling data and thus is not sensitive to the affinity and specificity of Ca^2+^ indicators. By allowing for the user to set custom look-back windows, *PeakCaller* is able to prevent type II errors and the rejection of a smaller peak in close proximity to a larger peak. Once the parameters have been set, *PeakCaller* automates the peak calling process for all regions of interest in the comma delimited (.csv) file, greatly increasing the efficiency of peak calling over manual peak calling.

Graphically, *PeakCaller* will denote a peak by placing a green circle around the point in both the original and the de-trended data. Should the user wish to compare their selected peak parameters and smoothing function to de-trended data using the “outstanding area” method of peak calling found in *PeakFinder*, the user can select the “show old peaks” box, which will display the original *PeakFinder* peaks using a red ‘X’.

### *PeakCaller*: visualization and connectivity

**Fig. 6:**
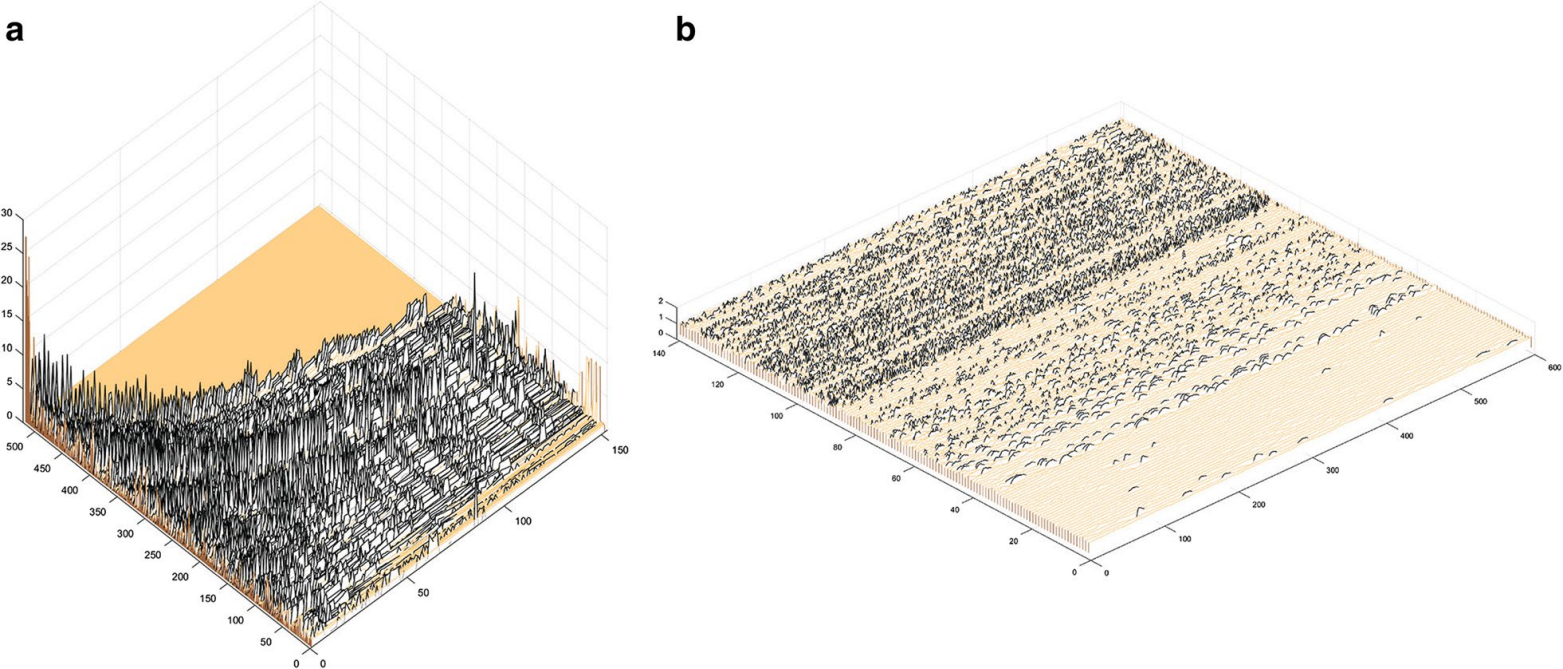
Multiple traces and ROIs can be visualized using the Peak Caller. The top dark parts of the trace are the identified peaks for each time series. This visualization can be rotated to examine the activity across an entire data set. **a** Ca^2+^ transients recorded from ~ 500 human iPSC-derived neurons transfected with the Ca^2+^ indicator GCamp6 recording using the ThermoFisher ArrayScan system. **b** Ca^2+^ transients recorded from ~ 200 human iPSC-derived neurons treated with the Ca^2+^ indicator Fluo-4 imaged at 10 Hz (x-axis represents time in seconds, y-axis represents number of ROIs and z-axis represents amplitude as ΔF/F)

In order to have a large scale visual representation of the traces in the context of the entire experiment, the view traces button can be used. Pressing the button labelled View Traces allows the user to see all of the fluorescence traces in a single figure, with the identified peaks highlighted in a different color. An example of this in a high content assay run across ~ 500 human iPSC-derived neurons expressing GCamp6 using the ThermoFisher ArrayScan and ~ 200 neurons treated with Fluo-4 and imaged at 10 Hz is presented in (Fig. 6). After visualization, the histograms button opens up two histograms: one summarizing the heights of the many peaks and the other the rise and fall times. These can be used to determine general characteristics of the peaks and help to get a population-based measurement of the transient behavior. To develop a general overview of the temporal clustering of Ca^2+^ transients, the end user can generate a raster plot. Pressing the button labelled Raster Plot allows the user to see a raster plot of all of the identified peaks in a single figure. This can be used as a way to easily visualize clusters of related activity overall in the data set.

To examine whether there is a functional relationship between ROIs, possibly determinant of neuronal connectivity, there are two types of measurements that *PeakCaller* offers. The first is by clicking the button labelled Correlations. *PeakCaller* then makes a run through all of the traces, looking pairwise for evidence of a relationship between each individual trace and the temporal shifts of the others. The results are then displayed graphically in a heat map. Note especially that the pairwise correlations computed here are based upon the original traces and not upon the software’s identification of individual peaks.

**Fig. 7:**
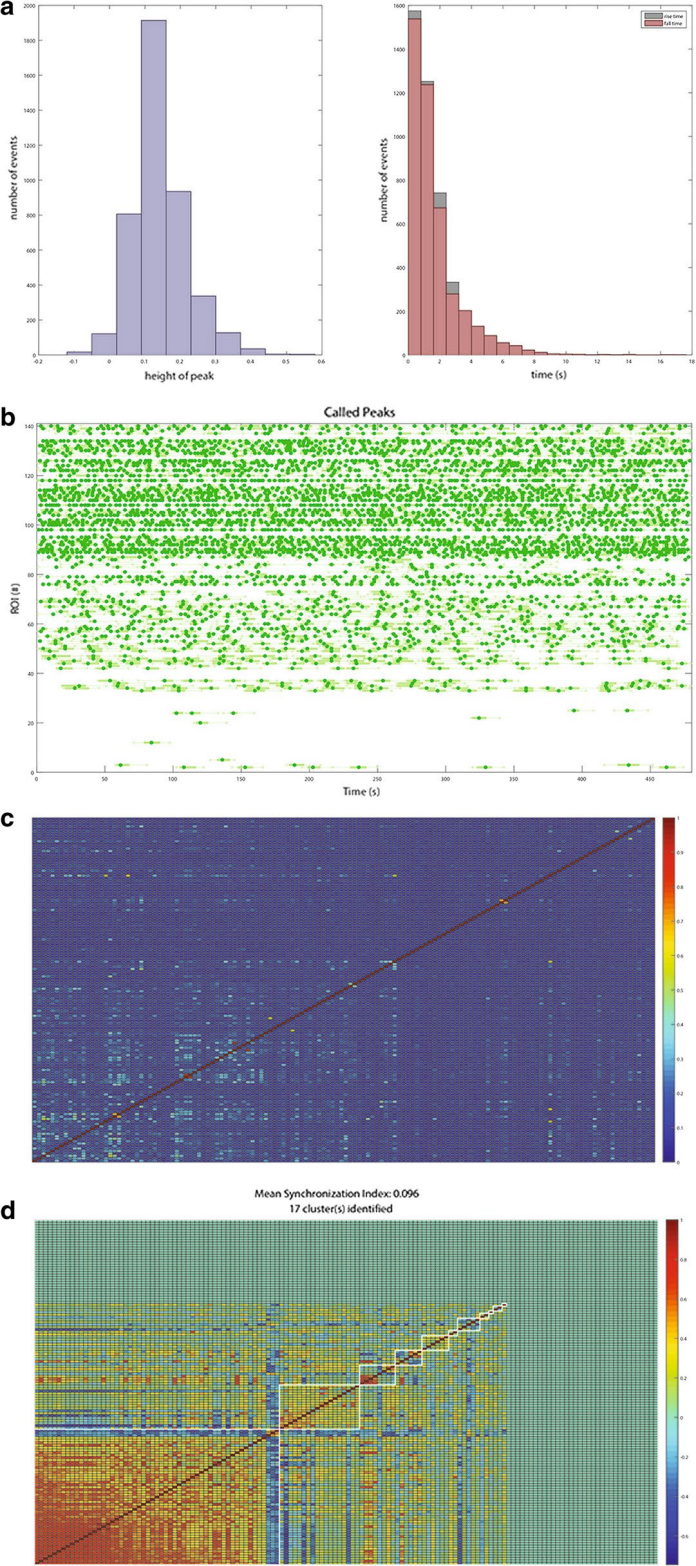
Typical functional output for a high-content Ca^2+^ imaging experiment. **a** Histograms, **b** raster plots, **c** correlation, and **d** synchronization indexes can be calculated for large numbers of cells to determine group characteristics of calcium transients in a high-throughput screen. The heat maps and synchronization index can give the end user a measure of the functional connectivity in a given dataset. In this dataset the Ca^2+^ transients are not functionally connected, as evidenced by no bursting patterns in the raster plot, low autocorrelations in the heat map, and a synchronicity index of 0.096. This is to be expected, as these transients were recorded from neural progenitors at day 21 derived from human iPSCs

Alternatively, a synchrony index can be constructed and calculated from the full set of ROIs. This index is computed by clicking the button labelled Synchronicity. In this case, the original traces are replaced with a bare-bones version that takes the value one near each peak (above the half-maximum value of the peak) and zero elsewhere. A generalized autocorrelation function is then produced for each of these traces, and a synchronization index is computed pairwise based on these autocorrelation functions. This method is similar to the one detailed in [25]. Because of the construction of the generalized autocorrelation functions, the pairwise indices should be the largest when the two ROIs have peaks that are similarly spaced, even if one series lags the other significantly. These pairwise values are then displayed graphically in a heat map. A second heat map is also displayed, with the ROIs rearranged in an attempt to group them into identifiable clusters. At the top of both heat maps, a “global” or “mean” index is displayed, representing the average index across all of the different pairs. This global/mean index should be closest to one if a large amount of pairwise synchrony is detected across all of the ROIs in the data set. This synchronization index, in combination with the correlations and the raster plot, can give the end user insight into whether the set of ROIs in the data set are functionally connected (Fig. 7).

## Results

**Fig. 8:**
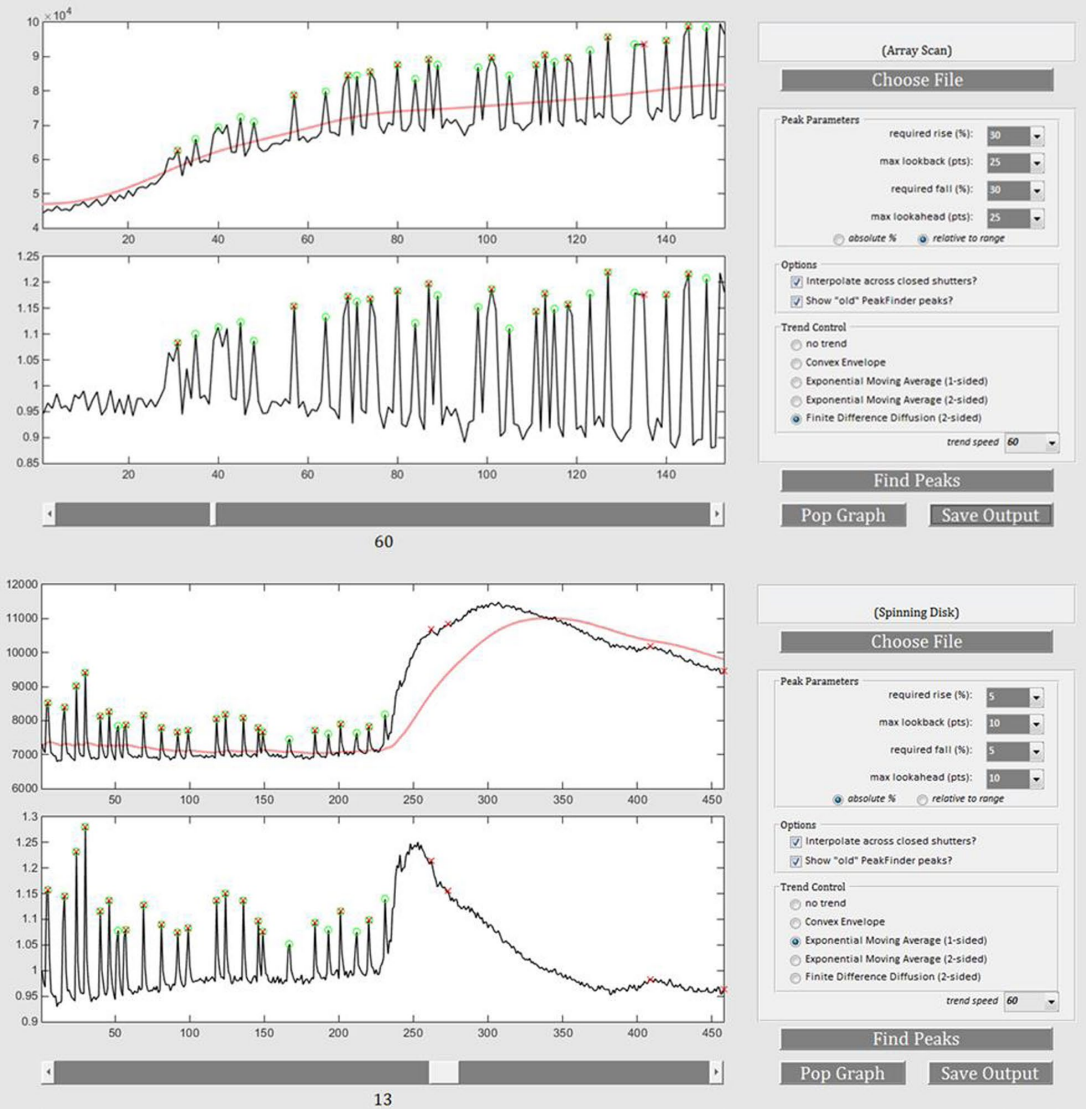
Analysis of Zeiss Spinning Disk and ThermoFisher Array Scan Ca^2+^ imaging data output by *PeakCaller*. The vertical axis represents Fluo-4 fluorescence emission intensity (arbitrary units) and the horizontal axis is time in seconds. Green circles represent *PeakCaller* identified peaks. Red Xs represent *PeakFinder* identified peaks. Parameters were chosen to best represent data generated on both platforms

**Fig. 9:**
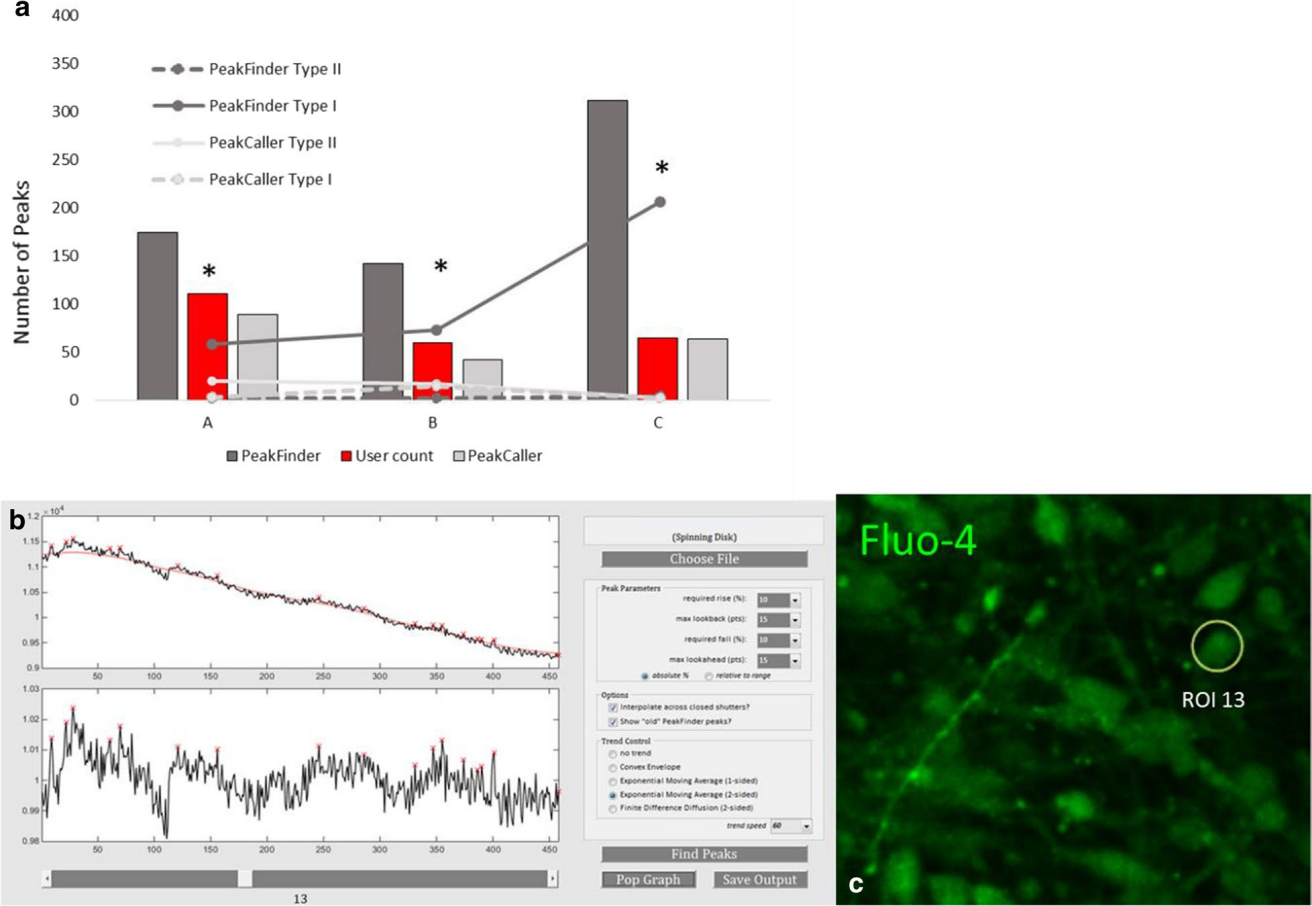
*PeakCaller* decreases the incidence of type I errors in cultured hiPSC-derived cortical neurons (*p < 0.05). **a** No difference in incidence of type II errors was found between *PeakCaller* and *PeakFinder* under these culture conditions. **b** Representative trace shows over estimation of peak incidence and frequency in *PeakFinder* (red X) script. **c** Designated ROI for the recording in this representative trace

In this study we use Fluo-4, a [Ca^2+^] indicator, and analyze Ca^2+^ signaling in commercially available hiPSC-derived cortical neurons and dissociated mouse cortical neuron cultures. Raw output files from the Zeiss Spinning Disk and ThermoFisher ArrayScan platforms were analyzed using *PeakCaller*. *PeakCaller* efficiently and accurately imported both data files and calculated the number of peaks per ROI (cell body) and peak intervals as shown in Fig. 8. Specific parameters were chosen for each file and analyzed accordingly. *PeakCaller* reduced the incidence of type I errors in day 30 cultured iPSC derived cortical neurons (*p* < 0.05) (Fig. 9) in three separate recordings. The incidence of type II errors was unaffected by the use of the *PeakCaller* script. The representative trace provided in Fig. 9 demonstrates how *PeakFinder* overestimates the number of peaks when presented with a signal that represents a high fluctuation in the background fluorescence. The *PeakFinder* algorithm uses an a priori approach that assumes that a peak is present, as it denotes a peak based on the highest point within a set window, and thus will always produce false positives in an instance where there is a background of fluorescence.

*PeakCaller* has several advantages over *PeakFinder* in these instances because the user can set a minimum rise and fall within a window (needed to denote a “peak”), as well as the width of the window to accurately represent the fluctuation of the data. The ability to accurately represent the frequency of Ca^2+^ signaling in developing neurons by reducing the instance of type I and type II errors is crucial to the identification of cellular phenotypes associated with neurological conditions and experimental manipulation. Once phenotypes associated with aberrant Ca^2+^ signaling have been identified, the program can be used to accurately measure the efficacy of pharmacological interventions. In Fig. 9, *PeakCaller* accurately identified 0 peaks in the ROI, while *PeakFinder* identified 17 false positive peaks.

Pharmacological manipulation is an important tool used to interrogate neuronal function. Picrotoxin (PTX) is a gamma-aminobutyric acid-alpha (GABA_A_) receptor antagonist that can induce rapid firing of action potentials and potentially glutamatergic excitotoxicity by significantly decreasing the activity of inhibition in neuronal circuits [26, 27]. After the application of PTX to cultured neurons, cells will enter a state of heightened excitability followed by loss of excitation after exhaustion of glutamate stores. This change in excitation and inhibition can be measured by looking at the activity of Ca^2+^ in neurons [27]. We exploited this function of PTX in two model systems in order to test the functionality of the *PeakCaller* script and compare it to *PeakFinder*.

**Fig. 10:**
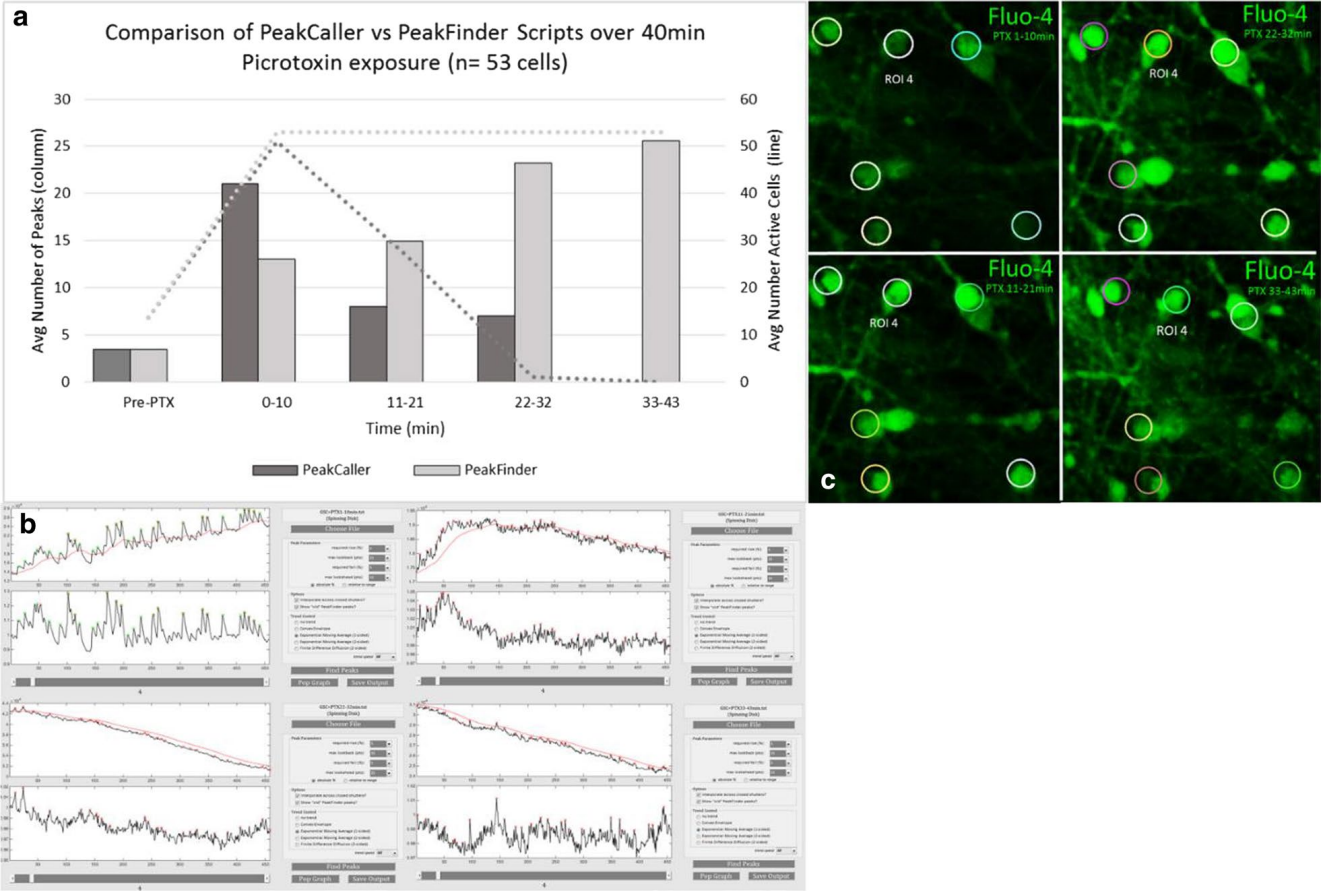
**a** Reduction of type I errors by *PeakCaller* after application of PTX to induce excitation. Prior to PTX exposure both *PeakFinder* and *PeakCaller* correctly identified the same number of peaks per active cell. After PTX exposure (50 μM), *PeakCaller* correctly modeled the state of induced excitation and excitotoxicity in the hiPSC derived cortical neurons, while *PeakFinder* initially under estimated the number of peaks and then over estimated the number or Ca^2+^ peaks. **b** Representative traces of ROI 4 are provided to show the transient increase in Ca^2+^ signaling after application of PTX. **c** Designated ROIs across all recordings for this representative experiment

To do this, we exposed d30 hiPSC-derived cortical neurons to 50 μM PTX with a 1 min wash in phase and 40 min recording (four 10 min recordings). The raw data was analyzed using both *PeakCaller* and *PeakFinder*. *PeakCaller* accurately identified windows of high Ca^2+^ activity in neurons, while *PeakFinder* consistently over-estimated the number of peaks and number of active cells (Fig. 10). In this context, *PeakFinder* becomes less accurate and more vulnerable to type I error as the number of true peaks decreases relative to background fluorescence.

The incidence of type I and type II errors in *PeakCaller*. *PeakFinder* scripts were also compared in the analysis of dissociated mouse cortical neurons. While a direct comparison of Ca^2+^ signaling in mouse and hiPSC-derived cortical neurons has not yet been published, it would be expected that these two cultures may have different Ca^2+^-transient characteristics.

**Fig. 11:**
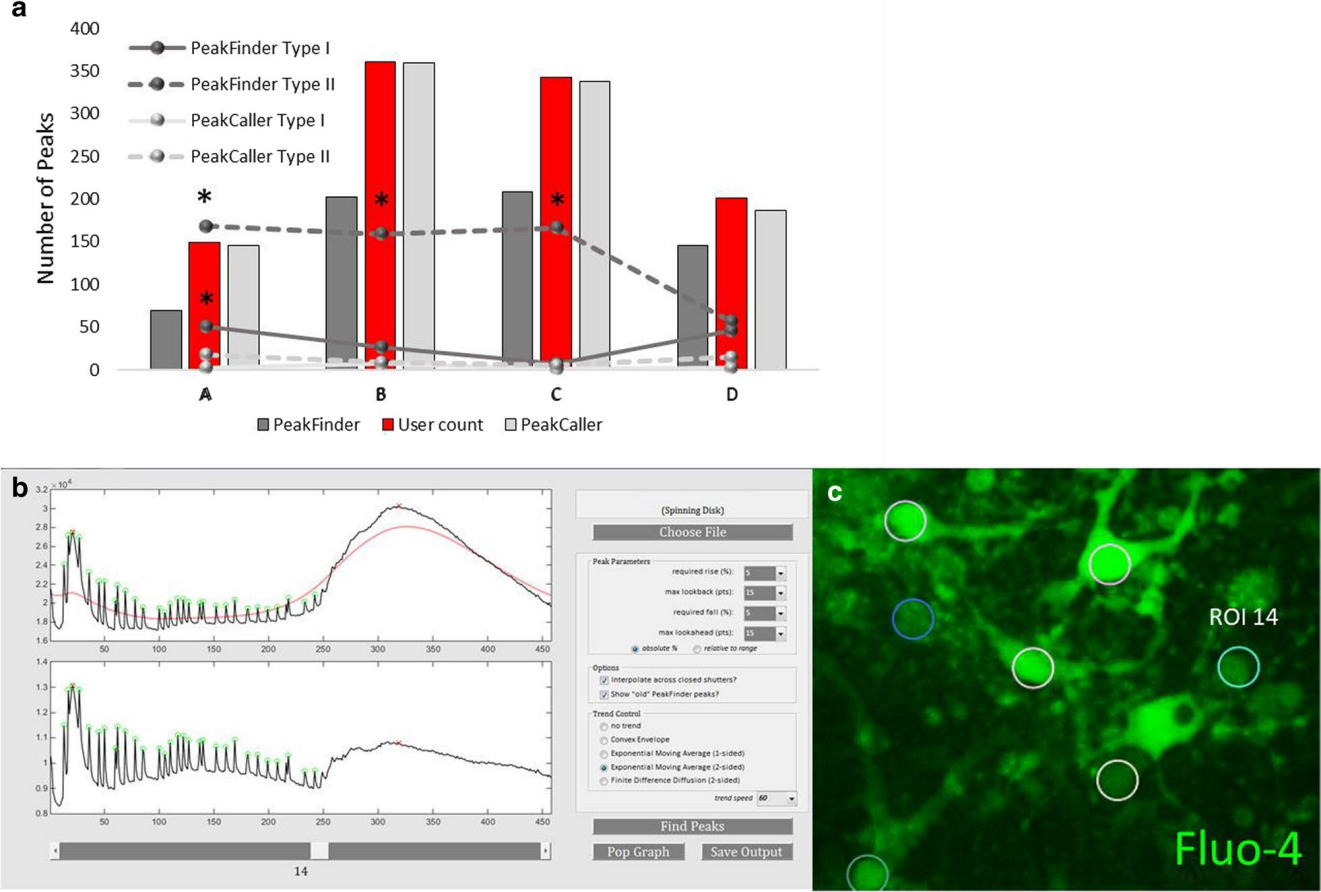
**a** Reduction of type II error in dissociated mouse cortical neurons. *PeakCaller* demonstrated the ability to reduce the occurrence of type II errors (*p* < 0.05) in three of four Ca^2+^ recordings. No difference in incidence of type II error was found between the two scripts for the fourth recording. *PeakCaller* also showed a significant (*p* < 0.05) reduction in type I error in recording A. **b** Representative traces below show *PeakCaller* peaks in green circles and *PeakFinder* peaks as red Xs. **c** Designated ROIs for this representative experiment

In four separate recordings, *PeakCaller* reduced the incidence of type II errors in three of the recordings. There was no difference in the incidence of type II errors in the fourth recording (Fig. 11). *PeakCaller* also indicated a significant (*p* < 0.05) reduction in type I error in recording A, but no difference in type I errors in the other recordings. In the representative trace in Fig. 4, *PeakFinder* identified only two peaks, whereas the user and *PeakCaller* identified 26 and 28 peaks, respectively. In this instance *PeakFinder* underestimated the number of peaks present for the same reason it over-estimated the number of peaks in the previous experiments. *PeakFinder* selected the two highest points in the data set as peaks and disregarded true peaks that fell below these higher points, due to *PeakFinder’s* inability to normalize the data set to changes in fluorescence. *PeakCaller*’s smoothing functions can be set to account for global changes in fluorescence and can be set with narrower look-back and look-ahead windows to properly identify the majority of peaks in a given ROI.

## Conclusions

Here we have demonstrated that *PeakCaller* can reduce the incidence of both type I and type II errors in hiPSC-derived cortical neurons, dissociated mouse cortical neurons, and cells in a state of induced neuronal excitation. *PeakCaller*’s advantages stem from two main features: (1) smoothing functions that can normalize changes in background fluorescence, and (2) settable parameters for minimum peak height and look-back/ahead windows. The script does not achieve perfect accuracy in its designation of Ca^2+^ peaks, but it significantly outperforms *PeakFinder* with respect to reduction in both types of statistical error. The fact that *PeakCaller* can reduce different types of error produced by different cell line characteristics and culture conditions is important to the study of neurodevelopmental, neuropsychiatric, and neurodegenerative diseases, particularly in the context of high-throughput screening. It is foreseeable that a disease condition may produce a phenotype that is more vulnerable to the significant type I or type II error bias introduced by the use of *PeakFinder*, and as such may confound any data analyzed using this algorithm.

*PeakCaller* allows the user freedom to designate peak parameters and smoothing functions to most accurately represent the raw data within a GUI that is accessible to users without extensive MATLAB or coding experience. Designed specifically to address the conditions/limitations of Ca^2+^ signaling in cultured neurons, *PeakCaller* is a powerful new tool in the hands of the neurophysiologist.

## Abbreviations

Ca^2+^: calcium
hiPSC: human induced pluripotent stem cell
ROI: region of interest
PTX: picrotoxin
GABA_A_: gamma-aminobutyric acid-alpha receptor
